# Flexibility Retained: Unimpaired Updating of Expectations in Schizophrenia

**DOI:** 10.3390/bs14010041

**Published:** 2024-01-07

**Authors:** Jian Li, Luo Chen, Dongsheng Zhou, Enze Tang, Jiewei Zheng, Xiaoqi Huang, Bao-Liang Zhong, Chenxiao Guan, Huiying Liu, Mowei Shen, Hui Chen

**Affiliations:** 1 Department of Psychology and Behavioral Sciences, Zhejiang University, Hangzhou 310030, China; 2 Ningbo Kangning Hospital, Ningbo 315200, China; 3 Department of Psychiatry, Wuhan Mental Health Center, Wuhan 430022, China

**Keywords:** expectation updating, predictive coding, cognitive flexibility, memory, attribute amnesia

## Abstract

Flexibly and actively updating expectations based on feedback is crucial for navigating daily life. Previous research has shown that people with schizophrenia (PSZ) have difficulty adjusting their expectations. However, there are studies suggesting otherwise. To explore this further, we used a novel trial-based expectation updating paradigm called attribute amnesia. In the task, the participants needed to report the location of a target stimulus among distractors in pre-surprise trials. In the surprise trial, they were unexpectedly asked to report the identity of the target before reporting its location. Afterward, control trials were conducted whereby the participants were asked the same questions as in the surprise trial. Notably, the surprise trial and control trials were nearly identical, except that the participants expected to be asked about identity information in the control trials but not in the surprise trial. Thus, an improvement in identity reporting accuracy in the control trials in comparison with the surprise trial indicated active updating of expectations. In the current study, a total of 63 PSZ and 60 healthy control subjects (HCS) were enrolled. We found that both the PSZ and the HCS were unable to report information that they had fully attended to (i.e., identity) in the surprise trial. However, both groups showed a significant improvement in reporting identity information even in the first control trial. Critically, there was no significant difference in the magnitude of improvement between the two groups. The current findings indicate that PSZ have the ability to update their expectations as quickly and flexibly as HCS, at least in the context of the current task. The possible factors that might contribute to the discrepancy regarding expectation updating are discussed.

## 1. Introduction

Expectations help us to navigate through daily events by shaping and influencing our anticipatory and reactive behaviors in various aspects of life. Evidence has revealed that expectation manipulation could possibly change the consequences of ambiguity perception [1], decision-making [2], risk-taking propensity [3], and even treatment efficacy [4]. However, in real-life scenarios, we sometimes come across occasions when the established expectations are violated by the incoming sensory input, and we may either maintain or update our prior expectations. In this sense, expectation updating, also referred to as belief updating, is defined as the adjustment of current higher-level predictions based on the receipt of new information [5]. Although the average person is capable of disregarding random noises and flexibly integrating the important violations facing disconfirmatory evidence, people with schizophrenia (PSZ) seem to inappropriately process expectation errors [6,7,8,9,10,11,12,13,14], probably leading to different patterns of expectation updating compared to the general population.

Inconsistent findings have been reported regarding the capability of PSZ to update their expectations. Empirical research suggests that PSZ are unable to appropriately adjust their expectations based on external information [6,7,8,9,10,11,12,13,14]. For example, in a simple perceptual task [15], participants were required to report the final direction of a random dot motion display, which sometimes changed direction midway through the trial. The results showed that the PSZ were significantly more likely to report the initial direction of the moving dots, even when the direction changed by 90 degrees. This suggests that PSZ have difficulty updating their beliefs even in the face of disconfirmatory evidence compared to healthy control subjects (HCS) [15]. In addition to the perceptual level [15,16], similar deficits have also been observed at the level of higher-order processes that involve evidence gathering [17,18,19], probability learning [20,21,22], and language processing [23,24]. It is also worth noting that this deficit in prediction updating was found to be associated with the severity of delusional symptoms in PSZ, indicating the potential interplay between belief updating and delusion development in schizophrenia [15]. 

Interestingly, there is opposite evidence suggesting that PSZ retain the ability to update their expectations in response to changing environments [25,26]. In one study, participants were asked to predict the tilt direction (left or right) of an upcoming visual stimulus, with probabilities (left vs. right) varying across blocks of the task. The results showed that PSZ were able to detect patterns and adjust their predictions based on different probabilities in a similar way to the HCS [25]. Another study provided further evidence that PSZ can update their expectations based on the changes in the context to optimize their performance once they have successfully learned the initial probabilistic discriminations [26]. The above-mentioned findings suggest that PSZ are capable of updating their expectations but may struggle in certain contexts and also propose the necessity to continue investigating the expectation updating capability in PSZ and its correlations with clinical symptoms.

Given the inconsistent findings in previous research, the present study aims to further investigate the ability of PSZ to update their expectations in response to contextual changes. Moreover, we used a trial-based expectation updating paradigm to investigate whether PSZ could update their expectations as instantly as HCS. 

## 2. Materials and Methods

### 2.1. Research Paradigm

Here, we adopted a novel paradigm called attribute amnesia [27], whereby the participants were repeatedly asked to report the location of a target stimulus (e.g., numbers larger than five) among three distractors (e.g., numbers smaller than five) and were then unexpectedly asked to report the identity of the target before reporting its location in a surprise trial. After this, several control trials were administered whereby the participants were asked the same questions as in the surprise trial. Research by Chen and Wyble [27,28] consistently demonstrated that college students were unable to report the target identity when they did not expect to do so, despite having used that identity to locate the target moments earlier. However, they were able to accurately report the identity information in the first control trial. Note that the surprise trial and control trials were essentially the same, with the exception that the participants anticipated the probe of identity information in the control trials but not in the surprise trial. Therefore, such an improvement in identity reporting tasks indicates active updating of expectations about what information should be probed. By comparing the performance in identity reporting tasks in the surprise and control trials in PSZ, we were able to directly examine their ability to flexibly update their expectations. In addition, the attribute amnesia task is straightforward and brief, with it only involving the localization of a target stimulus by its identity, and can be completed within a few minutes. This is particularly important considering that the performance of PSZ can be easily confounded by nonspecific factors, such as reduced motivation and poor task comprehension. Taken together, the attribute amnesia paradigm provides a valuable tool to investigate the ability of expectation updating in PSZ, and we hope to enhance our understanding of this important cognitive process in individuals with psychosis.

### 2.2. Participants 

We included two separate groups of participants, totaling 63 PSZ and 60 HCS. The initial results from the first group of participants have been preprinted and can be found on the OSF website [https://doi.org/10.31219/osf.io/szdx9 (accessed on 31 March 2023)]. To ensure the robustness and reliability of our findings, we conducted a replication experiment with the second group of participants. Remarkably, the results from the second group displayed precisely the same pattern as the first group. Therefore, for the sake of simplicity and clarity, we decided to combine and analyze the data from both groups in the current study. In the PSZ group, three of the PSZ were excluded from formal analysis due to low accuracy in the identity reporting task in the control trials, resulting in a final sample of 60 PSZ for formal analyses. All PSZ were clinically stable patients with a confirmed diagnosis of schizophrenia according to the International Classification of Diseases, tenth version (ICD-10) [29], by experienced psychiatrists. Symptoms and social function were assessed using the Positive and Negative Syndrome Scale (PANSS) [30] and the Social Disability Screening Schedule (SDSS) [31], respectively. The HCS were recruited through online and offline advertising. All participants, both the PSZ and HCS, reported normal or corrected-to-normal acuity and were free of other medical or neurologic comorbidities that could have affected their performance. The medication dosages of the PSZ were converted into chlorpromazine (CPZ) equivalents [32].

### 2.3. Stimuli and Procedure

The experiment was programmed and executed using MATLAB software R2014a (The MathWorks; Natick, MA, USA) with the Psychophysics Toolbox extension [33,34] and presented on a 14-inch laptop monitor (60 Hz, 1024 × 768 screen resolution). The participants sat at a viewing distance of approximately 50 cm. The background of the display was medium gray (RGB: 150, 150, 150).

**Figure 1:**
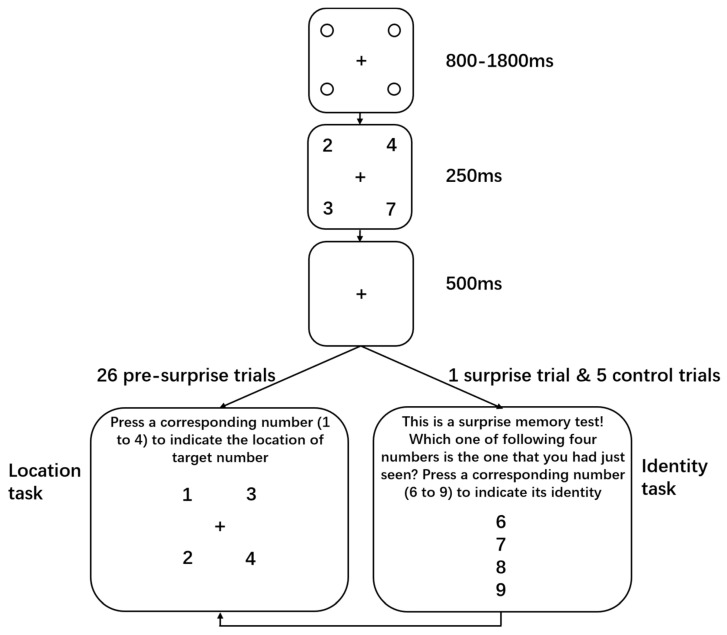
Trial sequence of the experiment. The participants were repeatedly asked to finish a target localization task in the pre-surprise trials, while they were prompted unexpectedly to complete a memory test in the surprise trial where they needed to indicate the identity of the target. The participants read the question and responded by pressing corresponding keys in the pre-surprise trials, whereas they gave verbal responses which were recorded by experimenters in the surprise trial and control trials. Note that the instructions on the display were actually presented in Chinese during the experiment.

As shown in Figure 1, Each trial began with a centered black fixation cross (0.57° visual angle) among four black placeholder circles (0.46°). The four placeholders were presented on the four corners of an invisible square (5.15° × 5.15°) centered on the screen. After a variable duration (800–1800 ms), the stimulus array appeared for 250 ms. The stimulus array contained one target number that was larger than five (6, 7, 8, or 9; 0.57° × 0.80°) and three distractor numbers (1 to 4; 0.57° × 0.80°), which were randomly presented at the four locations of the placeholders. This stimuli display was followed by a 500 ms fixation cross display. In the first 26 pre-surprise trials, the participants were asked to report the location where the target number had appeared (location task). In the twenty-seventh trial (i.e., the surprise trial), prior to the location task, the participants were unexpectedly presented with a forced-choice question requiring them to indicate which of four numbers was the target number they had just seen. Following the surprise trial, the participants completed five control trials which were in the same format as the surprise trial. The participants responded by pressing corresponding keys on a keyboard in the pre-surprise trials, whereas they gave verbal responses that were recorded by the experimenter in the surprise and control trials.

## 3. Results

Demographic data: Table 1 displays the participants’ characteristics. A total of 60 participants were included in the PSZ group (34 [57%] male and 26 [43%] female; mean [SD] age, 37.0 [10.2] years; mean [SD] education, 12.1 [2.6] years) and 60 participants were included in the HCS group (30 [50%] male and 30 [50%] female; mean [SD] age, 36.0 [10.3] years; mean [SD] education, 11.4 [3.3] years). The two groups were well matched for age (*p* = 0.606), gender (*p* = 0.189), and education (*p* = 0.464).

Pre-surprise performance: Both the HCS and the PSZ demonstrated high accuracy in correctly identifying the target number among the distractor numbers and reporting its location in the pre-surprise trials (HCS: 96%; PSZ: 95%), as summarized in Table 2. This suggested that both groups, including the PSZ, had a good understanding of the task.

Surprise trial performance: In the surprise trial, 30 out of 60 (50%) HCS and 25 out of 60 (42%) PSZ were able to correctly report the identity of the target number. Two groups showed comparable accuracy in reporting the target number’s identity: χ^2^(1, *N* = 120) = 0.839, *p* = 0.360, and φ = 0.08. Additionally, the two groups were similar in the location report task, with the HCS at 63% and the PSZ at 70%.

**Figure 2:**
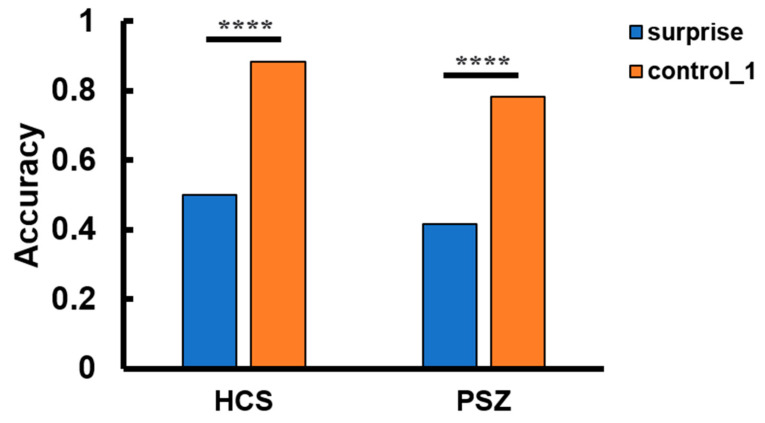
The accuracy in the identity reporting task in the surprise trial vs. the first control trial for the HCS (healthy control subjects) and the PSZ (people with schizophrenia). Both the HCS and the PSZ exhibited considerable improvement in reporting the identity information during the first control trial. **** *p* < 0.0001.

Control trials performance: As shown in Figure 2, the accuracy in the identity reporting task was significantly improved in the first control trial (i.e., the trial immediately following the surprise trial) compared to the surprise trial in both the HCS and PSZ; HCS: 88% vs. 50%, χ^2^ (1, *N* = 120) = 20.671, *p* < 0.0001, and φ = 0.42; PSZ: 78% vs. 42%, χ^2^ (1, *N* = 120) = 16.806, *p* < 0.0001, and φ = 0.37. Moreover, the PSZ showed comparable accuracy to the HCS in the first control trial (78% vs. 88%, χ^2^ (1, *N* = 120) = 2.16, *p* = 0.142, and φ = 0.13). These results indicated that the PSZ, like the HCS, were able to update their expectations based on the surprise question. Performance in the identity reporting task remained constant for the four remaining control trials for both groups (HCS: 92%, 93%, 95%, and 93%; PSZ: 92%, 93%, 95%, and 93%).

In terms of reporting location information, only the HCS showed significantly higher performance in the first control trial compared to the surprise trial, and this increase reached significance (83% vs. 63%, χ^2^ (1, *N* = 120) = 6.136, *p* = 0.013, and φ = 0.23), while the that of the PSZ did not (70% vs. 70%). However, the improvement did reach significance when comparing the surprise trial to the second control trial for the PSZ: 70% vs. 90%, χ^2^ (1, *N* = 120) = 7.5, *p* = 0.006, and φ = 0.25. The accuracy in the first control trial for the HCS was higher than for the PSZ (83% vs. 70%, χ^2^ (1, *N* = 120) = 2.981, *p* = 0.084, and φ = 0.16); this difference approached significance. Performance in the location reporting task remained constant in both groups for the three remaining control trials (HCS: 93%, 95%, and 98%; PSZ: 90%, 92%, and 85%).

Comparison of expectation updating between the two groups: Breslow–Day tests [35] were conducted to investigate whether there were any significant differences between the two groups in terms of performance improvement from the surprise trial to the first control trial. The results showed that there was no significant difference between the two groups, (χ^2^ = 0.412; *p* = 0.521), suggesting that the PSZ were able to update their expectations as swiftly and flexibly as the HCS.

Correlation analyses: To examine whether the demographic (e.g., age, education, and sex) and clinical (e.g., CPZ does equivalent, illness duration, PANSS subscale scores, and SDSS) variables were correlated with the performance of the PSZ, we calculated the correlation coefficients between these moderators and the accuracy of the identity report during the surprise trial and the first control trial and for the progress between these two trials. As our statistics encompassed an ordinal dependent variable and non-Gaussian distributed continuous independent variables, the non-parametric Spearman correlation index (rho) was used to reflect the correlational relationships. The results showed no significance of any bivariate correlations during the surprise trial or for the between-trial progress (all *p*-values > 0.05). However, in the first control trial, the PANSS positive scale score manifested a positive correlation with the accuracy of the identity report (rho = 0.261, *p* = 0.044), indicating that the PSZ with stronger positive symptoms tended to more successfully report identity information with instantly updated expectations. None of any of the other bivariate correlations during the first control trial reached significance (all *p*-values > 0.05).

## 4. Discussion

In this study, we used a trial-based paradigm to investigate whether PSZ could adjust their expectations in response to changes in context. We found that, like the HCS, the PSZ were unable to report information that they had fully attended to (i.e., identity) in a surprise trial. However, they showed a significant improvement in reporting identity information during the first control trial, indicating active updating of their expectations. Remarkably, this improvement occurred immediately in the first control trial, and no delay was observed when compared to the HCS. Our findings suggest that PSZ have a relatively intact ability to update their expectations, at least within the scope of the present task.

Previous research on expectation updating in PSZ has often utilized probability learning tasks, which usually involves the analysis of trial-averaged data to measure expectation updating [20,21,22,23]. As proposed by Broeker et al. [36], our study used a trial-based expectation updating paradigm that allowed us to examine updating based on a single trial, i.e., the surprise trial. Specifically, in this paradigm, the participants were given an expectation that only location information, but not identity information, needed to be memorized and reported in the pre-surprise trials. However, this expectation was violated in the surprise trial with the introduction of a new predictive message (i.e., the identity report) [37,38]. By comparing the accuracy of identity reporting between the control and surprise trials, we were able to determine whether and how quickly an update occurred due to the single surprise trial. One previous study modeled trial-by-trial expectation updating in PSZ [22]. However, the task used in the study was highly demanding in terms of working memory, and therefore, the results obtained might be complicated by impairments in their working memory [22]. In contrast, the task employed here was relatively easy, as it only required reporting information that had been used moments earlier. Thus, our findings may provide a relatively pure measure of expectation updating. 

Our study seems to contrast with a common-held belief that PSZ are characterized by preservation and inflexibility [39]. Indeed, there is a substantial amount of research suggesting that PSZ are impaired in appropriately updating their expectations [6,7,8,9,10,11,12,13,14]. However, there are also some studies suggesting otherwise [25,26]. Our study adds to this evidence by demonstrating that PSZ are actually capable of updating their expectations, at least in the context of the attribute amnesia task. One possible factor that may contribute to the discrepant findings is the general neurocognitive function of PSZ (e.g., working memory abilities) [22]. For example, research has shown that the difference in expectation updating between PSZ and HCS disappeared after removing the PSZ who performed poorly in basic neurocognitive tests and contingency discrimination from the analysis [26]. Furthermore, a study [25] that controlled for neurocognitive and demographic factors found no evidence of deficits in expectation updating among PSZ, suggesting that these factors may play a role in the ability of expectation updating. Another possible factor may be the experimental paradigm per se. The task used in this study was relatively simple and straightforward, as it solely involved updating expectations about whether earlier information needed reporting, without any contingency monitoring or probability learning as required in previous research [20,21,22,23]. Therefore, the current task might be more sensitive to reveal expectation updating, which could explain the reason as to why no deficits were observed in our study.

Our findings are illuminating for the impact of expectation on memory in schizophrenia. Specifically, correctly reporting the identity information in the first control trial required the participants to not only adjust their expectations accordingly but also successfully modulate their memory based on the updated expectations. Our findings indicated that the PSZ were able to memorize information according to their expectations, suggesting an unimpaired modulation of expectation on memory. Previous research has shown that PSZ exhibit disruptions in the modulation of expectations on perception [40], motor control [41,42], and language processing [43]. But relatively little is known concerning the impact of expectation on memory. One study investigating selective encoding in schizophrenia demonstrated that PSZ exhibited similar benefits to HCS in terms of memorizing items that were more likely to be tested compared to the less likely ones [44], which seemed to be consistent with our findings. However, their study was not originally designed to investigate the influence of expectation on memory and could not differentiate the effects of expectation and attention. In our study, the improvement in identity reporting accuracy is solely attributed to expectation updating. This is because the participants were required to fully attend to the identity of the target in both the surprise and control trials in order to locate it, with the only difference being whether they expected to report the identity information [45].

There are also some implications for the function of discarding outdated information in schizophrenia. Attribute amnesia is proposed to be elicited by the active removal of attended but outdated information (i.e., information that individuals fully attended to but expect not to report in the near future) [46]. An attribute amnesia task typically requires participants to locate a target stimulus by its identity (e.g., numbers larger than five) without reporting the identity information [27]. In this context, individuals must first identify the identity of the target to locate it. After successfully locating the target, they actively discard the identity information because they expect it to be unnecessary for the subsequent location reporting task [46]. Our study found that PSZ also exhibited the attribute amnesia phenomenon, so it seemed that they may not necessarily have deficits in filtering irrelevant information [47]. Alternatively, research has shown that PSZ tend to focus more intensely on a subset of information (i.e., hyperfocusing), which leads to greater filtering of irrelevant information (i.e., hyperfiltering) [48]. Therefore, PSZ may actually struggle with actively filtering outdated information (i.e., the identity) [49], but they simultaneously displayed hyperfocusing on relevant information (i.e., the location) and the hyperfiltering of irrelevant information [48] in the current task. In other words, the observed low accuracy in the identity report in the surprise trial was due to the hyperfiltering of identity rather than active inhibition/filtering.

In the subsequent correlation analyses, only one significant positive correlation between the positive symptom scale and identity report accuracy was identified in the first control trial, suggesting that more severe positive symptoms were associated with stronger instant expectation updating. According to the predictive coding account of psychosis [6], this population has been characterized by reduced precision of prior beliefs and increased precision of the upcoming sensory data, and their perceptual inference abnormally relies more on prediction errors. This was reflected by the higher accuracy of the identity report (i.e., prediction error) and the lower accuracy of the location report (i.e., prior belief) in the first control trial among the PSZ in general compared to the surprise trial. It is then reasonable to infer that such a tendency for the improved identity report was more prominent among more typical PSZ with stronger positive symptoms, such as hallucinations and delusions. In fact, the correlation identified here corroborates previous research results [16], but more future research is needed to continue examining the correlations between clinical measures and prediction updating performance.

One limitation of our study is that all PSZ are medicated and chronic patients. It is therefore unknown whether the current findings can be generalized to first-episode patients who are not medicated [50]. Furthermore, our research reported an intact ability in expectation updating among PSZ; more research is warranted for a better understanding of the mechanism underlying expectation updating in this population.

## 5. Conclusions 

Through a straightforward and brief task, the current study demonstrates that PSZ are able to update their expectations as flexibly and instantly as HCS, suggesting that their ability to update expectations is relatively intact.

## Figures and Tables

**Table 1:** Participants’ characteristics (mean (SD)).

Demographics	PSZ (*n* = 60)	HCS (*n* = 60)	Statistics	
Age, y	37.0 (10.2)	36.0 (10.3)	*t* = −0.517	*p* = 0.606
Education, y	12.1 (2.6)	11.4 (3.3)	*t* = −1.322	*p* = 0.189
No. male/female	34:26	30:30	χ^2^ = 0.536	*p* = 0.464
Duration of illness, y	14.4 (9.1)			
CPZ does equivalent, mg/d ^1^	371.9 (187.2)			
PANSS	56.0 (17.5)			
Positive scale	15.5 (6.5)			
Negative scale	13.4 (6.1)			
General psychopathology scale	27.1 (8.6)			
SDSS ^2^	9.2 (4.1)			

Note: PSZ, people with schizophrenia; HCS, healthy control subjects; PANSS = Positive and Negative Syndrome Scale; SDSS = Social Disability Screening Schedule; CPZ = chlorpromazine. ^1^ Data missing for six PSZ; ^2^ Data missing for one person with schizophrenia.

**Table 2:** The accuracy results of the HCS and the PSZ.

	Pre-Surprise	Surprise	Control_1	Control_2	Control_3	Control_4	Control_5
Identity							
HCS		50%	88%	92%	93%	95%	93%
PSZ		42%	78%	92%	93%	95%	93%
Location							
HCS	96%	63%	83%	82%	93%	95%	98%
PSZ	95%	70%	70%	90%	90%	92%	85%

Note: PSZ, people with schizophrenia; HCS, healthy control subjects.

## Data Availability

Data are available from the corresponding author upon reasonable request.

## References

[1] Leong Y.C., Hughes B.L., Wang Y., Zaki J. (2019). Neurocomputational mechanisms underlying motivated seeing. Nat. Hum. Behav..

[2] Rungratsameetaweemana N., Itthipuripat S., Salazar A., Serences J.T. (2018). Expectations Do Not Alter Early Sensory Processing during Perceptual Decision-Making. J. Neurosci..

[3] Jepma M., Schaaf J.V., Visser I., Huizenga H.M. (2022). Impaired learning to dissociate advantageous and disadvantageous risky choices in adolescents. Sci. Rep..

[4] Bingel U., Wanigasekera V., Wiech K., Ni Mhuircheartaigh R., Lee M.C., Ploner M., Tracey I. (2011). The effect of treatment expectation on drug efficacy: Imaging the analgesic benefit of the opioid remifentanil. Sci. Transl. Med..

[5] Kube T., Rozenkrantz L. (2021). When Beliefs Face Reality: An Integrative Review of Belief Updating in Mental Health and Illness. Perspect. Psychol. Sci..

[6] Sterzer P., Adams R.A., Fletcher P., Frith C., Lawrie S.M., Muckli L., Petrovic P., Uhlhaas P., Voss M., Corlett P.R. (2018). The Predictive Coding Account of Psychosis. Biol. Psychiatry.

[7] Adams R.A., Stephan K.E., Brown H.R., Frith C.D., Friston K.J. (2013). The computational anatomy of psychosis. Front. Psychiatry.

[8] Ford J.M., Mathalon D.H. (2012). Anticipating the future: Automatic prediction failures in schizophrenia. Int. J. Psychophysiol..

[9] Fletcher P.C., Frith C.D. (2009). Perceiving is believing: A Bayesian approach to explaining the positive symptoms of schizophrenia. Nat. Rev. Neurosci..

[10] Horga G., Abi-Dargham A. (2020). An integrative framework for perceptual disturbances in psychosis. Nat. Rev. Neurosci..

[11] Thakkar K.N., Diwadkar V.A., Rolfs M. (2017). Oculomotor Prediction: A Window into the Psychotic Mind. Trends Cogn. Sci..

[12] Corlett P.R., Horga G., Fletcher P.C., Alderson-Day B., Schmack K., Powers A.R. (2019). Hallucinations and Strong Priors. Trends Cogn. Sci..

[13] Heinz A., Murray G.K., Schlagenhauf F., Sterzer P., Grace A.A., Waltz J.A. (2019). Towards a Unifying Cognitive, Neurophysiological, and Computational Neuroscience Account of Schizophrenia. Schizophr. Bull..

[14] Jardri R., Denève S. (2013). Circular inferences in schizophrenia. Brain.

[15] Bansal S., Bae G.Y., Robinson B.M., Hahn B., Waltz J., Erickson M., Leptourgos P., Corlett P., Luck S.J., Gold J.M. (2022). Association Between Failures in Perceptual Updating and the Severity of Psychosis in Schizophrenia. JAMA Psychiatry.

[16] Weilnhammer V., Röd L., Eckert A.L., Stuke H., Heinz A., Sterzer P. (2020). Psychotic Experiences in Schizophrenia and Sensitivity to Sensory Evidence. Schizophr. Bull..

[17] So S.H., Siu N.Y., Wong H.L., Chan W., Garety P.A. (2016). ‘Jumping to conclusions’ data-gathering bias in psychosis and other psychiatric disorders—Two meta-analyses of comparisons between patients and healthy individuals. Clin. Psychol. Rev..

[18] Speechley W.J., Whitman J.C., Woodward T.S. (2010). The contribution of hypersalience to the “jumping to conclusions” bias associated with delusions in schizophrenia. J. Psychiatry Neurosci..

[19] Sanford N., Veckenstedt R., Moritz S., Balzan R.P., Woodward T.S. (2014). Impaired integration of disambiguating evidence in delusional schizophrenia patients. Psychol. Med..

[20] Voss M., Moore J., Hauser M., Gallinat J., Heinz A., Haggard P. (2010). Altered awareness of action in schizophrenia: A specific deficit in predicting action consequences. Brain.

[21] Horga G., Schatz K.C., Abi-Dargham A., Peterson B.S. (2014). Deficits in predictive coding underlie hallucinations in schizophrenia. J. Neurosci..

[22] Nassar M.R., Waltz J.A., Albrecht M.A., Gold J.M., Frank M.J. (2021). All or nothing belief updating in patients with schizophrenia reduces precision and flexibility of beliefs. Brain.

[23] Sharpe V., Weber K., Kuperberg G.R. (2020). Impairments in Probabilistic Prediction and Bayesian Learning Can Explain Reduced Neural Semantic Priming in Schizophrenia. Schizophr. Bull..

[24] Brown M., Kuperberg G.R. (2015). A Hierarchical Generative Framework of Language Processing: Linking Language Perception, Interpretation, and Production Abnormalities in Schizophrenia. Front. Hum. Neurosci..

[25] Kreis I., Zhang L., Moritz S., Pfuhl G. (2021). Spared performance but increased uncertainty in schizophrenia: Evidence from a probabilistic decision-making task. Schizophr. Res..

[26] Reddy L.F., Waltz J.A., Green M.F., Wynn J.K., Horan W.P. (2016). Probabilistic Reversal Learning in Schizophrenia: Stability of Deficits and Potential Causal Mechanisms. Schizophr. Bull..

[27] Chen H., Wyble B. (2015). Amnesia for object attributes: Failure to report attended information that had just reached conscious awareness. Psychol. Sci..

[28] Chen H., Wyble B. (2016). Attribute amnesia reflects a lack of memory consolidation for attended information. J. Exp. Psychol. Hum. Percept. Perform..

[29] World Health Organization (1992). The ICD-10 Classification of Mental and Behavioural Disorders: Clinical Descriptions and Diagnostic Guidelines.

[30] Kay S.R., Fiszbein A., Opler L.A. (1987). The positive and negative syndrome scale (PANSS) for schizophrenia. Schizophr. Bull..

[31] Cooper J.E., Sartorius N. (1996). Mental Disorders in China: Results of the National Epidemiological Survey in 12 Areas.

[32] Leucht S., Samara M., Heres S., Davis J.M. (2016). Dose Equivalents for Antipsychotic Drugs: The DDD Method. Schizophr. Bull..

[33] Brainard D.H. (1997). The Psychophysics Toolbox. Spat. Vis..

[34] Kleiner M., Brainard D., Pelli D. (2007). What’s new in psychtoolbox-3. Perception.

[35] Breslow N.E., Day N.E. (1980). Statistical Methods in Cancer Research Volume I: The Analysis of Case-Control Studies.

[36] Broeker L., Kiesel A., Aufschnaiter S., Ewolds H.E., Gaschler R., Haider H., Künzell S., Raab M., Röttger E., Thomaschke R. (2017). Why Prediction Matters in Multitasking and How Predictability Can Improve It. Front. Psychol..

[37] Chen H., Yu J., Fu Y., Zhu P., Li W., Zhou J., Shen M. (2019). Does attribute amnesia occur with the presentation of complex, meaningful stimuli? The answer is, “it depends”. Mem. Cognit..

[38] Fu Y., Yan W., Shen M., Chen H. (2021). Does consciousness overflow cognitive access? Novel insights from the new phenomenon of attribute amnesia. Sci. China Life Sci..

[39] Zhu C., Kwok N.T., Chan T.C., Chan G.H., So S.H. (2021). Inflexibility in Reasoning: Comparisons of Cognitive Flexibility, Explanatory Flexibility, and Belief Flexibility Between Schizophrenia and Major Depressive Disorder. Front. Psychiatry.

[40] Keane B.P., Silverstein S.M., Wang Y., Papathomas T.V. (2015). Reduced depth inversion illusions in schizophrenia are state-specific and occur for multiple object types and viewing conditions. J. Abnorm. Psychol..

[41] Ford J.M., Mathalon D.H., Heinks T., Kalba S., Faustman W.O., Roth W.T. (2001). Neurophysiological evidence of corollary discharge dysfunction in schizophrenia. Am. J. Psychiatry.

[42] Shergill S.S., White T.P., Joyce D.W., Bays P.M., Wolpert D.M., Frith C.D. (2014). Functional magnetic resonance imaging of impaired sensory prediction in schizophrenia. JAMA Psychiatry.

[43] Curcic-Blake B., Liemburg E., Vercammen A., Swart M., Knegtering H., Bruggeman R., Aleman A. (2013). When Broca goes uninformed: Reduced information flow to Broca’s area in schizophrenia patients with auditory hallucinations. Schizophr. Bull..

[44] Gold J.M., Fuller R.L., Robinson B.M., McMahon R.P., Braun E.L., Luck S.J. (2006). Intact attentional control of working memory encoding in schizophrenia. J. Abnorm. Psychol..

[45] Chen H., Yan N., Zhu P., Wyble B., Eitam B., Shen M. (2019). Expecting the unexpected: Violation of expectation shifts strategies toward information exploration. J. Exp. Psychol. Hum. Percept. Perform..

[46] Fu Y., Zhou Y., Zhou J., Shen M., Chen H. (2021). More attention with less working memory: The active inhibition of attended but outdated information. Sci. Adv..

[47] Luck S.J., Leonard C.J., Hahn B., Gold J.M. (2019). Is Attentional Filtering Impaired in Schizophrenia?. Schizophr. Bull..

[48] Luck S.J., Hahn B., Leonard C.J., Gold J.M. (2019). The Hyperfocusing Hypothesis: A New Account of Cognitive Dysfunction in Schizophrenia. Schizophr. Bull..

[49] Eich T.S., Nee D.E., Insel C., Malapani C., Smith E.E. (2014). Neural correlates of impaired cognitive control over working memory in schizophrenia. Biol. Psychiatry.

[50] Xu Y.M., Deng F., Zhong B.L. (2022). Facial emotion identification impairments in Chinese persons living with schizophrenia: A meta-analysis. Front. Psychiatry.

